# Supplementary material for: Dissection of Common Rust Resistance in Tropical Maize Multiparent Population through GWAS and Linkage Studies
Source: Plants (Basel). 2024 May 18;13(10):1410. doi: 10.3390/plants13101410 (PMC11125173; doi:10.3390/plants13101410)
Supplement: Supplementary file 1 [file plants-13-01410-s001.zip › plants-2904514-supplementary.pdf]

Supplementary Table S1. QTLs located in three RIL populations.

BLUP

Pop	QTL	Chr	Position(cM)	Mapping Interval(cM)	left marker	right marker	LOD	Additive_Effect	R^2 (%)
CML312	qRUST3-1	3	103.72	101.71-103.72	S3_21,766,539	S3_19,468,979	3.92	-0.59	0.1
CML312	qRUST3-2	3	106.73	105.73-108.28	S3_18,118,650	S3_17,098,052	3.17	-0.54	0.08
CML312	qRUST6-1	6	36.39	36.39-38.39	S6_108,339,246	S6_99.941.104	4.71	0.92	0.12
D39	qRUST2-1	2	28.49	25.05-31.32	S2_125,535,857	S2_125,535,857	4.71	-0.48	0.09
D39	qRUST3-3	3	54.96	54.41-59.02	S3_210,543,887	S3_172,823,884	5.39	0.7	0.11
Y32	qRUST4-1	4	40.43	40.12-43.27	S4_128,564,645	S4_121,288,117	3.37	0.46	0.08
Y32	qRUST4-2	4	52.39	51.39-53.39	S4_94,866,787	S4_94,866,787	3.1	0.45	0.08

21JH

CML312	qRUST6-2	6	36.73	36.39-38.39	S6_110,692,870	S6_99.941.104	5.21	1.08	0.13
D39	qRUST3-4	3	54.96	54.41-59.59	S3_210,543,887	S3_172,823,884	6.62	0.8	0.13
D39	qRUST3-5	3	96.21	96.21-96.21	S3_212,366,601	S3_212,366,601	3.1	-0.4	0.06
					**21YS**				
CML312	qRUST1-1	1	58.36	58.36-58.36	S1_81,924,769	S1_81,924,769	3.24	0.64	0.08
CML312	qRUST1-2	1	65.58	64.58-65.59	S1_87,398,960	S1_87,398,960	3.04	0.62	0.07
CML312	qRUST3-6	2	117.03	117.03-117.03	S2_14,177,571	S2_14,177,571	3.1	-0.83	0.08
D39	qRUST2-2	2	28.49	28.49-29.32	S2_125,535,857	S2_125,535,857	3.26	-0.48	0.07
D39	qRUST3-7	3	55.51	54.41-56.51	S3_210,543,887	S3_172,823,884	3.62	0.68	0.07
Y32	qRUST4-3	4	54.83	51.39-55.45	S4_94,866,787	S4_63,985,272	3.56	0.59	0.08
Y32	qRUST4-4	4	58.92	57.39-63.39	S4_57,388,250	S4_28,135,211	4.77	0.68	0.11
					**22YS**				

CML312	qRUST8-1	8	24.93	24.93-24.93	S8_91,109,417	S8_91,109,417	3.07	0.82	0.07
D39	qRUST2-3	2	28.49	25.17-32.32	S2_125,535,857	S2_125,535,857	4.94	-0.61	0.1
Y32	qRUST4-2	4	53.39	51.39-53.39	S4_94,866,787	S4_94,866,787	3.4	0.49	0.08

Supplementary Table S2. Common rust significant SNP set.

**Environment**	**SNP**	**Chr**	**POS**	**REF**	**ALT**	**MAF**
BLUP	snp-54614501	2	54614501	A	G	0.094311
BLUP	snp-164385099	2	164385099	A	G	0.396254
BLUP	snp-203116317	3	203116317	A	G	0.162442
BLUP	snp-203116453	3	203116453	T	C	0.160959
BLUP	snp-204202469	3	204202469	A	G	0.163934
BLUP	snp-224639688	3	224639688	T	G	0.163102
BLUP	snp-122025827	4	122025827	T	C	0.082589
BLUP	snp-122025836	4	122025836	C	T	0.082222
BLUP	snp-122025839	4	122025839	A	C	0.082405
BLUP	snp-122025849	4	122025849	T	A	0.083146
BLUP	snp-118608561	5	118608561	T	C	0.411557
BLUP	snp-118608571	5	118608571	C	A	0.410165
BLUP	snp-118608579	5	118608579	G	C	0.411557
BLUP	snp-76107442	6	76107442	C	T	0.155136
BLUP	snp-76107445	6	76107445	G	T	0.154812
BLUP	snp-118876904	8	118876904	G	C	0.14405
BLUP	snp-102507767	10	102507767	G	T	0.16557

21JH	snp-54614501	2	54614501	A	G	0.094311
21JH	snp-200524370	2	200524370	T	C	0.181472
21JH	snp-104441686	3	104441686	C	T	0.065903
21JH	snp-203116453	3	203116453	T	C	0.160959
21JH	snp-204202469	3	204202469	A	G	0.163934
21JH	snp-221066619	3	221066619	G	A	0.165533
21JH	snp-224639688	3	224639688	T	G	0.163102
21JH	snp-118608561	5	118608561	T	C	0.411557
21JH	snp-118608571	5	118608571	C	A	0.410165
21JH	snp-118608579	5	118608579	G	C	0.411557
21JH	snp-55178561	6	55178561	G	A	0.075163
21JH	snp-55178563	6	55178563	C	G	0.075163
21JH	snp-76107442	6	76107442	C	T	0.155136
21JH	snp-76107445	6	76107445	G	T	0.154812
21JH	snp-61591553	7	61591553	C	G	0.477564
21JH	snp-118876904	8	118876904	G	C	0.14405
21JH	snp-102507767	10	102507767	G	T	0.16557
21YS	snp-54614501	2	54614501	A	G	0.094311
21YS	snp-164385099	2	164385099	A	G	0.396254
21YS	snp-212692082	5	212692082	C	T	0.173697
21YS	snp-53643137	7	53643137	C	T	0.109756
21YS	snp-118876904	8	118876904	G	C	0.14405
21YS	snp-102507767	10	102507767	G	T	0.16557
22YS	snp-54614501	2	54614501	A	G	0.094311
22YS	snp-206814920	2	206814920	A	C	0.116176

22YS	snp-206814979	2	206814979	G	A	0.116279
22YS	snp-206815067	2	206815067	G	A	0.104816
22YS	snp-203116317	3	203116317	A	G	0.162442
22YS	snp-203116453	3	203116453	T	C	0.160959
22YS	snp-203116471	3	203116471	C	T	0.159453
22YS	snp-204202469	3	204202469	A	G	0.163934
22YS	snp-220956352	3	220956352	C	T	0.166065
22YS	snp-221066619	3	221066619	G	A	0.165533
22YS	snp-224029612	3	224029612	C	T	0.442623
22YS	snp-224029629	3	224029629	T	C	0.468927
22YS	snp-224029717	3	224029717	G	A	0.433333
22YS	snp-224639688	3	224639688	T	G	0.163102
22YS	snp-122025816	4	122025816	T	A	0.084283
22YS	snp-122025827	4	122025827	T	C	0.082589
22YS	snp-122025836	4	122025836	C	T	0.082222
22YS	snp-122025839	4	122025839	A	C	0.082405
22YS	snp-122025850	4	122025850	A	T	0.082589
22YS	snp-54530619	5	54530619	T	C	0.019774
22YS	snp-118608571	5	118608571	C	A	0.410165
22YS	snp-74247937	6	74247937	A	T	0.3775
